# Research Progress on Antioxidant Peptides from Fish By-Products: Purification, Identification, and Structure–Activity Relationship

**DOI:** 10.3390/metabo14100561

**Published:** 2024-10-20

**Authors:** Xinru Liu, Qiuyue Hu, Yafang Shen, Yuxin Wu, Lu Gao, Xuechao Xu, Guijie Hao

**Affiliations:** 1 School of Food Science and Engineering, Yangzhou University, Yangzhou 225127, China; mz220230823@stu.yzu.edu.cn (X.L.);; 2 Key Laboratory of Healthy Freshwater Aquaculture, Ministry of Agriculture and Rural Affairs, Key Laboratory of Fish Health and Nutrition of Zhejiang Province, Huzhou Key Laboratory of Aquatic Product Quality Improvement and Processing Technology, Zhejiang Institute of Freshwater Fisheries, Huzhou 313001, China

**Keywords:** oxidative stress, amino acid types, molecular weight, structure–activity, fish by-product, purification and identification

## Abstract

**Background/Objectives:** Excessive reactive oxygen species (ROS) can lead to oxidative stress, which has become an urgent problem requiring effective solutions. Due to the drawbacks of chemically synthesized antioxidants, there is a growing interest in natural antioxidants, particularly antioxidant peptides. **Methods**: By reviewing recent literature on antioxidant peptides, particularly those extracted from various parts of fish, summarize which fish by-products are more conducive to the extraction of antioxidant peptides and elaborate on their characteristics. **Results**: This article summarizes recent advancements in extracting antioxidant peptides from fish processing by-products, Briefly introduced the purification and identification process of antioxidant peptides, specifically focusing on the extraction of antioxidant peptides from various fish by-products. Additionally, this article comprehensively reviews the relationship between amino acid residues that compose antioxidant peptides and their potential mechanisms of action. It explores the impact of amino acid types, molecular weight, and structure–activity relationships on antioxidant efficacy. **Conclusions**: Different amino acid residues can contribute to the antioxidant activity of peptides by scavenging free radicals, chelating metal ions, and modulating enzyme activities. The smaller the molecular weight of the antioxidant peptide, the stronger its antioxidant activity. Additionally, the antioxidant activity of peptides is influenced by specific amino acids located at the C-terminus and N-terminus positions. Simultaneously, this review provides a more systematic analysis and a broader perspective based on existing research, concluded that fish viscera are more favorable for the extraction of antioxidant peptides, providing new insights for the practical application of fish by-products. This could increase the utilization of fish viscera and reduce the environmental pollution caused by their waste, offering valuable references for the study and application of antioxidant peptides from fish by-products.

## 1. Introduction

Severe oxidative stress can cause significant damage to organisms, as reactive oxygen species (ROS) attack membrane phospholipids, proteins, and nucleic acids [1]. Currently, the food, livestock farming, and cosmetics industries frequently utilize synthetic antioxidants to mitigate ROS. These antioxidants mainly include tert-Butylhydroxyanisole (BHA) and Butylated hydroxytoluene (BHT) [2]. Synthetic antioxidants are cost-effective and exhibit high efficacy; however, they are also associated with certain toxicological concerns and adverse side effects [3]. Antioxidant peptides possess the ability to effectively eliminate ROS and inhibit lipid peroxidation. Antioxidant peptides primarily clear ROS by donating hydrogen atoms and chelating metal ions. Compared to synthetic antioxidants, antioxidant peptides exhibit strong biological activity [4]. Their simple molecular structure and low molecular weight facilitate easier absorption by the body [5], and they can stably exert their function without exhibiting immunotoxicity.

As one of the three major animal-based foods alongside livestock and poultry meat and eggs, fish and aquatic products play a vital role in ensuring human nutrition. The processing by-products of freshwater fish, a high-quality resource, are rich in various natural active substances such as collagen and peptides, which have been proven to alleviate oxidative stress. The recycling and utilization of their nutritional and functional components present broad application prospects [6]. This article mainly summarizes the characteristics of antioxidant peptides and the extraction of antioxidant peptides from fish processing by-products. This work underscores the importance of sustainable practices in the fishery industry by promoting the efficient use of fish processing by-products. Existing research on extracting antioxidant peptides from fish by-products has primarily focused on fish meat or fish skin, potentially without analyzing which type of fish by-product is more suitable for extracting antioxidant peptides. This review clearly shows that the antioxidant effect of visceral extracts is superior, which can provide direction for researchers in selecting research subjects. Previous articles have placed more emphasis on introducing bioactive peptides, with antioxidant properties being only briefly introduced, or they have introduced antioxidant peptides themselves or a few known sequences of antioxidant peptides, without deeply summarizing and analyzing the antioxidant peptides extracted from fish. This article systematically analyzes and summarizes the relationship between the activity and structure of antioxidant peptides extracted from different fish by-products, and in terms of the activity of antioxidant peptides, it does not only focus on the characteristics of the peptides themselves, such as the impact of molecular weight, amino acid composition, and structure on the level of antioxidant activity, but also links their structure to the mechanism of antioxidant action, in order to promote in-depth research on antioxidant peptides extracted from fish by-products and provide a reference for their further development and utilization. By doing so, it opens avenues for further research and development in the field of natural antioxidants, contributing to advancements in nutrition, health, and environmental conservation.

## 2. Antioxidant Peptides Derived from Fish By-Products

Exogenous antioxidant peptides, crucial for neutralizing free radicals and mitigating oxidative stress, are derived from various biological sources, including plants, animals, and microorganisms. According to the data released in “The State of World Fisheries and Aquaculture 2024: Blue Transformation in Action” by the Food and Agriculture Organization of the United Nations, global fishery and aquaculture production surged to a record high of 223.2 million tons in 2022. Both freshwater fish and marine fish produce a large amount of by-products during the processing. Globally, more than 20 million tons of fish tissue are discarded annually, accounting for approximately 70% of the total weight of fish [7]. Fish processing generates by-products like scales, skin, heads, viscera, and bones, all of which are nutrient-dense. Fish and fish products are recognized for their highly digestible proteins and nutritional excellence [8]. Fish muscle is a high-quality source of antioxidant peptides. Jia et al. [9] extracted antioxidant peptides from the grass carp muscle. However, using fish muscle for this purpose is not optimal. Fish muscle is better consumed directly or used in dishes for its nutritional value, and extracting peptides from it may waste this value. Therefore, fish by-products are more suitable for extracting functional components. Freshwater fish processing by-products, like peptides, collagen, fish oil, and polysaccharides, are valuable resources. This approach addresses waste disposal, protects the environment, and fully utilizes the fish’s value. Antioxidant peptides, a type of polypeptide, have been extensively studied in fish by-products. This section summarizes recent studies on extracting antioxidant peptides from various fish by-products (Table 1). As shown in the table, these peptides contain hydrophobic amino acids, have a molecular weight of approximately 1000 Da, and function to scavenge free radicals.

### 2.1. Antioxidant Peptides Extracted from Fish Scales

Fish scales are bony derivatives formed from collagen in the dermis through long-term evolution. They contain proteins, ash, fats, gums, various vitamins, zinc, iron, calcium, phosphorus, and other essential trace elements, polyunsaturated fatty acids, and lecithin. The main components of fish scales are hydroxyapatite and protein. Proteins make up 50% to 70% of the total mass of fish scales, mainly consisting of collagen and keratin, with a small amount of globulin. The extraction rate of procollagen from the scales of tropical freshwater carp is 13.6%. The amino acid sequence of the purified collagen is very similar to that of European carp collagen, containing 18 amino acids [21]. Given their composition and value, fish scales are more suitable for extracting collagen.

### 2.2. Antioxidant Peptides Extracted from Fish Skin

**Figure 1:**
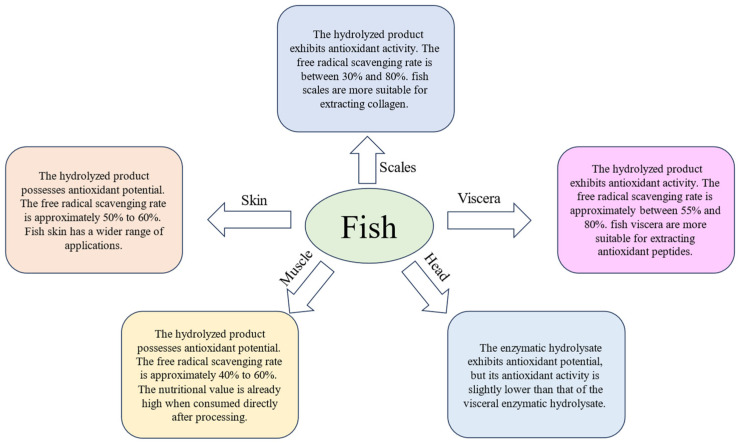
Fish by-product analysis diagram (the data in the chart is summarized based on Table 1).

Fish skin accounts for approximately 8% to 10% of the total weight of fish [22]. It is mainly composed of high-molecular-weight elastin, connective tissue protein, and collagen and can serve as an important source of high-value-added compounds such as collagen, gelatin, lipase, and bioactive peptides [23]. Due to diseases and the dietary restrictions of certain religious groups regarding poultry meat, extracting collagen from fish skin has become the preferred choice. Fish skin is rich in collagen and other bioactive components, which can be converted into antioxidant peptides through specific extraction and processing procedures, The free radical scavenging rate is approximately 50% to 60% (Figure 1). Fish skin can not only be used to extract functional components but also to make ready-to-eat foods such as puffed fish skin and fish skin jelly [24,25]. Additionally, it can be utilized in the manufacturing of various types of clothing. Compared to other fish waste products, fish skin has a wider range of applications. However, considering factors such as extraction processes, cost-effectiveness, and market demand, fish skin may not necessarily be the best resource among fish processing waste for extracting antioxidant peptides.

### 2.3. Antioxidant Peptides Extracted from Fish Head

The fish head accounts for 9% to 12% of the total weight of the fish and is rich in high-quality protein. Fish heads are a potential source for peptide industrialization with good yields. Islam et al. [26] extracted peptides from sturgeon heads with a higher total yield than skin collagen peptide and skin tissue peptide. The percentage of peptides with a molecular weight of less than 500 Da in head tissue peptide was significantly higher, resulting in stronger antioxidant activity. The free radical scavenging rate is approximately 40% to 60% (Figure 1). The hardness of fish heads and bones necessitates specialized processing techniques, increasing the difficulty of extraction.

### 2.4. Antioxidant Peptides Extracted from Fish Viscera

As the main by-product of fish processing, fish viscera account for approximately 10% of the fish body mass [27]. The amino acid composition of the proteins found in fish internal organs is comprehensive, including essential amino acids such as lysine, methionine, and threonine, which are required by the human body. The amino acid pattern of its protein is relatively balanced. Therefore, fish intestines and viscera can be used as a good source of protein production. Ganesh et al. [28] used a combination of pepsin and trypsin to hydrolyze the intestines and viscera of gray mullet fish. After separation and purification, they obtained an antioxidant peptide with a molecular weight of 701.9 Da and a sequence of Ala-Met-Thr-Gly-Leu-Glu-Ala. At a concentration of 1 mg/mL, the DPPH scavenging rate reached 54%, and the metal chelating ability reached 78.6%. Je et al. [29] obtained protein hydrolysates through enzymatic hydrolysis of tuna liver using different proteases, which exhibited high reducing power, DPPH scavenging ability, and other properties, indicating strong antioxidant activity. Compared to fish scales, heads, and bones, which are tough and difficult to process, fish skin offers diversified applications. In terms of weight proportion, processing methods, nutritional content, and usage scenarios, fish viscera are more suitable for extracting antioxidant peptides.

## 3. The Separation and Purification of Antioxidant Peptides

The bioactivity of bioactive peptides is determined by their properties, such as molecular weight, charge, and hydrophobicity. Therefore, the separation and purification of bioactive peptides are fundamental to the identification of their activity and structure, and appropriate separation and purification methods are usually selected based on the different properties of peptides, such as molecular weight, polarity, and isoelectric point. Separation methods for peptides based on molecular weight include ultrafiltration (UF) [27], nano-ultrafiltration (NF), and gel filtration [30]. Ion-exchange chromatography separates peptides based on their charge, while reversed-phase high-performance liquid chromatography (RP-HPLC) separates bioactive peptides based on their hydrophobicity and hydrophilicity [31]. The initial step in antioxidant peptide purification typically involves membrane separation techniques, which can be categorized based on pore size into nanofiltration, ultrafiltration, and microfiltration. In subsequent purification steps, gel filtration chromatography and ion-exchange chromatography are the most commonly used methods. For instance, Dong et al. [32] separated two peptide fractions from the hydrolysate of shiitake mushroom protein using Sephadex G-15 gel filtration chromatography and preliminarily determined the molecular weights of these fractions to be 2385 Da and 1138 Da based on standard samples. Reversed-phase high-performance liquid chromatography (RP-HPLC) is the final purification step, with its separation principle based on the varying hydrophobicity of peptide mixtures with the stationary phase, eluting weakly hydrophobic peptide fractions first and then strongly hydrophobic ones. This method boasts advantages such as good stability, high separation efficiency, and broad applicability, but it is more suitable for separating small peptides, and hence, it is often used in conjunction with other techniques. Jang et al. [33] first purified peptide fractions using Sephadex G-25 gel filtration chromatography based on their molecular weights, then obtained purer peptides using strong cation-exchange chromatography based on charge characteristics, and finally collected the effective ACE-inhibiting peptides from the enriched mushroom using RP-HPLC technology.

After a series of preparation and purification steps, it is necessary to identify the structure and function of the obtained antioxidant peptides. The most commonly used method for peptide structure identification is mass spectrometry. The principle of mass spectrometry involves using a mass spectrometer to bombard peptides into ionic fragments, which are then classified and analyzed based on their different mass-to-charge ratios. Typically, the obtained peptide subfractions are compared with protein databases to obtain the specific amino acid sequences. Due to the high efficiency, sensitivity, and good reproducibility of mass spectrometry, it has been widely used for peptide sequence identification. In addition, mass spectrometry is often combined with liquid chromatography (LC/MS), leveraging the separation capabilities of liquid chromatography and the grouping capabilities of mass spectrometry. The functional activity is determined based on the final amino acid sequence and composition of the peptide.

## 4. The Relationship between the Structure of Antioxidant Peptides and Their Activity

In living organisms, maintaining a balance between oxidants and antioxidants is crucial for redox homeostasis. When this balance is disrupted, it leads to oxidative stress, which can cause significant oxidative damage to the body. Such damage includes DNA damage, lipid peroxidation, and protein damage [34]. Antioxidant peptides play a vital role in mitigating these effects by scavenging free radicals and reactive oxygen species or by preventing oxidative damage through the inhibition of the lipid peroxidation chain reaction [35]. Antioxidant peptides are bioactive peptides with antioxidant activity, generally consisting of 2 to 20 amino acids. Their antioxidant activity is believed to be closely related to their amino acid composition, sequence, and molecular weight. We will discuss the structure–activity relationship in this section.

### 4.1. The Relationship between Antioxidant Activity and Amino Acid Residue Types or Sequence

The composition of amino acids within the peptide is one of the most important factors for the antioxidant activity of antioxidant peptides [4]. For instance, Agrawal et al. [36] extracted antioxidant peptides from pearl millet, which contain a significant number of hydrophobic amino acids, including glycine (Gly), leucine (Leu), and proline (Pro). Similarly, five novel peptide sequences, FSAP (Phe-Ser-Ala-Pro), PVETVR (Pro-Val-Glu-Thr-Val-Arg), QEPLLR (Gln-Glu-Pro-Leu-Leu-Arg), EAAY (Glu-Ala-Ala-Tyr), and VLRPPLS (Val-Leu-Arg-Pro-Pro-Leu-Ser), were identified from Paeonia Ostii “Feng Dan” hydrolysate [37]. Two antioxidant peptides prepared from Tilapia were identified and the amino acid sequences were identified as Asp-Cys-Gly-Tyr and Asn-Tyr-Asp-Glu-Tyr [38]. A common characteristic of these peptide sequences is the presence of one or more hydrophobic or aromatic amino acids, which contribute to their antioxidant activity [39]. Notably, the hydroxyl radical scavenging ability of Trp-Pro-Pro is greater than that of Gln-Pro, highlighting the importance of specific amino acid compositions in antioxidant efficacy. The hydroxyl radical scavenging ability of Trp-Pro-Pro is greater than that of Gln-Pro. Matsui et al. [40] demonstrated that amino acids with hydrophobic residues, such as alanine, valine, leucine, isoleucine, proline, phenylalanine, and methionine, have strong antioxidant activity against peroxyl radicals by measuring the myoglobin protection ratio. During the hydrolysis process, the peptide bond at the C-terminus of hydrophobic amino acids breaks, and after cleavage, the hydrophobic amino acids are located at the terminal group of the new peptide chain, where they can exert their antioxidant effects [41]. Hydrophobic amino acids in polypeptides can eliminate free radicals and are related to antioxidant activity [42]. The sulfhydryl group of the cysteine residue not only possesses strong electron-donating capability but also has significant hydrogen-donating ability [43]. Due to the presence of a hydroxyl group on its aromatic ring, Tyr can provide electrons to radicals, thereby quenching them [44]. Amino acids containing a benzene ring can provide protons to free radicals, thereby slowing down the chain reaction of free radicals [45]. As shown in Table 1, antioxidant peptides extracted from fish by-products consistently exhibit free radical scavenging properties. Notably, peptides extracted from fish viscera demonstrate that the presence of hydrophobic amino acid residues positively impacts the antioxidant activity of these peptides.

The thiol group of cysteine (Cys), the imidazole group of histidine (His), the indole group of tryptophan (Trp), and the hydroxyl group of threonine (Thr) can all bind to metal ions, thereby exerting antioxidant activity [46]. Additionally, aspartic acid (Asp) and lysine (Lys) residues can deactivate the pro-oxidant activity of metal ions [47]. The imidazole ring of histidine, in particular, is known to chelate metal ions, thus inhibiting metal ion-catalyzed oxidation reactions [36]. In a study of the antioxidant activity of porcine myofibrillar protein peptides, Saiga et al. [47] found that under higher pH conditions, acidic amino acid residues form complexes with metal ions, thereby inhibiting lipid peroxidation. Furthermore, the residues of glycine (Gly), alanine (Ala), valine (Val), leucine (Leu), isoleucine (Iso), and proline (Pro) improve the solubility of antioxidant peptides in lipids through their fatty side chains, enhancing their antioxidant effects [48].

The chelation of antioxidant peptides with metal ions can prevent the formation of free radicals and exert antioxidant effects by altering the chemical reactivity of the metals, forming insoluble metal complexes, or spatially hindering the interaction between metals and lipids. This mechanism of action is also observed in some antioxidant peptides extracted from fish by-products. For instance, the antioxidant peptide YGDEYS, extracted from tilapia skin, exerts its antioxidant effect by chelating metal ions through its amino acid residues glutamic acid (Glu) and aspartic acid (Asp). Similarly, polypeptides extracted from horse mackerel skin can also chelate metal ions, contributing to their antioxidant activity (Table 1).

The amino acids composing antioxidant peptides can not only exert antioxidant effects by scavenging free radicals and chelating metal ions but also achieve antioxidant effects by regulating ROS oxidase. The interaction between the amino acid residues of antioxidant peptides and ROS-producing oxidases reduces the generation of ROS, thereby preventing oxidation [49]. For instance, tryptophan (Trp) interacts with the key residues of xanthine oxidase (XO) and molybdopterin MOS3004, thus inhibiting XO activity [50]. As shown in Table 2, the polypeptide extracted from seahorse can promote the expression of antioxidant enzymes by regulating signaling pathways, thereby protecting LO2 cells from oxidative damage. Additionally, other polypeptides listed in the table can exert their antioxidant activities by enhancing the activities of catalase, superoxide dismutase, and glutathione peroxidase.

Antioxidant peptides extracted from fish by-products may also directly interact with enzymes, either inhibiting or enhancing their activities or regulating the expression levels of related proteins to alleviate oxidative stress. The amino acid composition of antioxidant peptides significantly contributes to their antioxidant activity. When several peptides contain the aforementioned specific amino acid residues, yet their antioxidant activities differ, it indicates that antioxidant activity is not only related to the type of amino acid residues but may also be influenced by the sequence of the peptides. For example, the activities of preferred sequences were found to be Glu-Leu (EL) > Tyr-Phe-Tyr-Pro-Glu-Leu (YFYPEL) > Phe-Tyr-Pro-Glu-Leu (FYPEL) > Tyr-Pro-Glu-Leu (YPEL) > Pro-Glu-Leu (PEL), indicating that the EL sequence is crucial for antioxidant activity [51].

**Table 2:** The research status of in vivo experiments on antioxidant peptides.

Source	Amino Acid Sequence	Antioxidant Property (In Vivo)	Characteristics	References
Green odorous frog (*Odorrana margaratae*) skin	GLLSGHYGRASPVAC	The peptide reduced the levels of lipid peroxidation and malondialdehyde and protected epidermal cells from UVB-induced apoptosis by inhibiting DNA damage via down-regulation of p53, caspase-3, caspase-9, and Bax and up-regulation of Bcl-2.	DPPH RSA: 5~20%; ABST^+^ RSA: 5~90%; Fe^3+^ reduction capacity:1~7%	[52]
Oyster (*Crassostrea hongkongensis*)	LTDDQVDEIIRN LTDDQVDEIIRNT LTDDQVDEIIR MWEGEEPTPSEGGPTPK WEGEEPTPSEGGPTPK NNDDIEGSPFK SIDVVILDPH	OPs treatment promoted antioxidant enzyme (SOD and GPH-Px) activities and decreased malondialdehyde (MDA) level in the skin. OPs protected against UVB-induced skin photodamage by virtue of its antioxidative and anti-inflammatory properties, as well as regulating the abnormal expression of MMP-1. The possible molecular mechanism underlying OPs anti-photoaging is possibly related to down-regulating of the MAPK/NF-κB signaling pathway, while promoting TGF-β production in the skin.	molecular weights (<2000 Da): 95.6%	[53]
Hemiscorpius lepturus	YLYELR AFPYYGHHLG	HL-7 (927.30 Da) exhibits higher antioxidant activity than HL-10 (1161.41 Da) in preventing the decline of CAT and SOD activities in the serum and liver of mice induced by D-galactose.	activity of CAT in the D-galactose-treated group (2.57 mK/mg protein)	[54]
Walnut	TNPSDSAPGTIR EDFGGGHPDPN MLPHHKDAES- VAVVTRG RAT HFREGDVIAFPAGVAH DIVAIPAGVAH VREIREGDVVAIPAGVAH LVYIEQGEGLLGL ATGEGFEWVSFK LPHHKDAESVAVVTRGRAT RGDIVAIPAGVAH REGDVIAFPAGVAH GLRGEEMEEMVQSA EGDIIAFPAGVAH FMLPHHKDAESVAVVTRGRAT	The increase in SOD and GSH-Px activities and the decrease in MDA levels in the liver and serum tissues of mice indicate that the polypeptide exerts good antioxidant activity in vivo.	DPPH RSA: 76.31 ± 1.52%;	[55]
Apostichopus japonicus	HEPFYGNEGALR	Increasing antioxidant enzyme activities in mice with acute alcoholic liver injury. A 20 mg/kg peptide supplement could activate the Nrf2/HO-1 pathway and block the nuclear translocation of NF-κB to alleviate oxidative stress and inflammation		[56]
Large yellow croaker (*Pseudosciaena crocea*)		The protein hydrolysate of large yellow croaker can stimulate the increase in the activities of superoxide dismutase, catalase, glutathione peroxidase, and glutathione reductase, lead to an elevation in the level of reduced glutathione, and reduce the level of malondialdehyde.		[57]
Whey protein		Enhancing the activities of catalase, superoxide dismutase, and glutathione peroxidase and reducing the level of malondialdehyde provide protective effects against d-galactose-induced aging.		[58]
Whole-grain Qingke (Tibetan *Hordeum vulgare* L.)	WGCQ WGPQ	Reducing the fasting plasma glucose, malondialdehyde, reactive oxygen species, plasma cortisol, and brain amyloid beta peptide (1–42) levels and increasing the superoxide dismutase and glutathione peroxidase activities, plasma dehydroepiandrosterone and 5-hydroxytryptamine levels, and brain gamma-aminobutyric acid contents of DT-mice		[59]
Seahorse (Hippocampus)	PAGPRGPA	Restoring the superoxide dismutase (SOD) and glutathione (GSH) levels and attenuating ethanol-induced oxidative damage and inflammation. Regulating the Kelch-like ECH-associated protein 1 (Keap1)/nuclear factor erythroid 2-related factor 2 (Nrf2) signaling pathway to protect LO2 cells from oxidative damage by promoting the expression of antioxidant enzymes	IC_50_ values (DPPH): 367.63 μg m L^−1^ (SBP), 6.03 μg m L^−1^ (Trolox); IC_50_ values (ABTS): 467.38 μg m L^−1^ (SBP), 6.65 μg m L^−1^ (Trolox);	[60]
Horse mackerel (*Magalaspis cordyla*)	ACFL	When peptides control free radicals, the chain reaction from superoxide to hydrogen peroxide does not occur in the presence of SOD and CAT, thereby restoring the activities of catalase and superoxide dismutase.		[61]

### 4.2. The Relationship between Antioxidant Activity and Amino Acid Position

The position of specific amino acids in peptide chains is crucial to their antioxidant properties. The N-terminus of antioxidant peptides is often composed of hydrophobic amino acids, while the C-terminus is mostly hydrophilic amino acids. Hydrophobic amino acids at the N-terminus possess aliphatic hydrocarbon side chains, which help antioxidant peptides migrate from the cell membrane to the reactive oxygen species (ROS) site for scavenging reactions, effectively inhibiting lipid peroxidation [62]. On the other hand, antioxidant peptides with hydrophilic groups at the C-terminus can improve their solubility, thus enhancing bioavailability [63]. The amino acid residues near the C-terminus not only possess hydrophilic properties but also exhibit hydrogen bond properties. Additionally, both the amino acid residues at the C-terminal and N-terminal possess electronic properties, with the C-terminal electronic properties contributing significantly to antioxidant activity [64].

Existing research employs computer-aided molecular models to further demonstrate the significance of amino acid types and positions in relation to antioxidant activity. For instance, techniques such as molecular docking and quantitative structure–activity relationship (QSAR) analyses are used to screen and predict the potential activities of peptides

Similarly, W. Li et al. [65] conducted research on the structure–activity relationship of antioxidant peptides (P1–P5) from watermelon seeds using quantum chemistry and molecular docking methods. They discovered that P1 can stably enhance the stability of Keap1 through interactions with active sites Asn382, Arg380, and Tyr334. Wang et al. [66] found that the glutamic acid residue of the antioxidant peptide from soft-shelled turtle (EDYGA) can bind to the arginine residue of Keap1 thus inhibiting Nrf2 degradation.

Furthermore, Chen et al. [67] performed hydrolysis of walnut protein to obtain three antioxidant peptides: HGEPGQQQR, VAPFPEVFGK, and HNVADPQR. Through molecular docking analysis, the results indicated that these three peptides can effectively bind to Keap1, CYP2E1, and TLR4 proteins. Zhang et al. [68] investigated the potential antioxidant stress mechanism of walnut polypeptides on HT22 cells through molecular docking. All six polypeptides successfully docked with Keap1, and EYWNR and FQLPR exhibited strong binding forces at the Nrf2 binding site within the Keap1-Kelch domain. Specifically, Keap1 formed twelve hydrogen bonds with EYWNR, including six key residues (Arg380, Arg415, Arg 483, Ser 363, Ser602), two hydrophobic interactions involving a key residue (Arg415), and a salt bridge containing a key residue (Arg415). Meanwhile, Keap1 formed eleven hydrogen bonds with FQLPR, including two key residues (Arg380, Ser555) and three hydrophobic interactions including one key residue (Tyr334). Therefore, these peptides seem to directly inhibit the Keap1-Nrf2 interaction, leading to the release of free Nrf2, demonstrating the good inhibitory effect of antioxidative peptides on neural cells. To investigate the antioxidant effects of tuna peptides, molecular docking analysis was performed between the peptide AGLYPGA, which had the highest predicted score, and the Keap1 protein. The results indicated that it has a stable binding capacity with Keap1, potentially regulating the Keap1/Nrf2-ARE signaling pathway and thereby enhancing antioxidant levels [69]. Based on the examples provided, antioxidant peptides can modify the cellular oxidative stress response by regulating the Keap1-Nrf2 pathway. Antioxidant peptides extracted from fish by-products may also have this function.

These studies illustrate that the antioxidant activity of peptides is influenced by the specific amino acids present at the C-terminal and N-terminal positions. For example, peptides with tyrosine (Tyr) at both termini demonstrate high hydroxyl radical scavenging activity, as seen in antioxidant peptides from tilapia skin. These findings suggest that the antioxidant properties of peptides, particularly those extracted from fish by-products, are closely related to the presence and positioning of specific amino acids at the termini.

### 4.3. The Relationship between Antioxidant Activity and Molecular Weight

The molecular weight of peptides affects antioxidant activity. Oligopeptides containing 2 to 10 amino acids have higher antioxidant activity than most other peptides or proteins [70]. Most peptides with antioxidant activity have a molecular weight of less than 6000 Da. Antioxidant peptides with molecular weights ranging from 0.5 to 3 kDa tend to have stronger antioxidant activities [71]. Chen et al. [72] used alkaline protease to hydrolyze the golden sunflower seed protein to prepare antioxidant peptides. Then, they subjected the enzymatic hydrolysate to ultrafiltration, and the results showed that the peptides with a molecular weight of less than 3 kDa exhibited the strongest antioxidant capacity. Xia et al. [73] investigated the antioxidant activity of mung bean hydrolysate (MPH) and separated it into two main fractions (MPH-1 < 3 kDa and MPH-2 > 3 kDa) through ultrafiltration. They found that the purified MPH-1 fraction exhibited the highest antioxidant activity. Aondona et al. [74] subjected the sesame protein hydrolysate to ultrafiltration, which resulted in fractions with peptide sizes of <1 kDa, 1–3 kDa, 3–5 kDa, and 5–10 kDa. They found that the free radical scavenging and metal ion chelating abilities were significantly enhanced after ultrafiltration, and the <1 kDa peptide fraction exhibited the highest activity. Antioxidant peptides with lower molecular weights are more likely to exert biological effects by passing through the intestinal barrier, thus enhancing their bioavailability and therapeutic potential [75]. Both in vivo and in vitro experiments have demonstrated that lower molecular weight correlates with higher antioxidant activity (see Table 1 and Table 2). Therefore, antioxidant peptides with low molecular weight are considered more effective. Research consistently shows that peptides with lower molecular weights possess stronger antioxidant activities. This characteristic makes them promising candidates for developing functional foods and pharmaceuticals aimed at mitigating oxidative stress.

## 5. Summary and Prospects

Firstly, this article provides a comprehensive overview of the influence of amino acid residue types on the mechanism of antioxidant peptides, including their direct scavenging of free radicals, chelating metal ions, and reducing the levels of ROS-producing oxidases. It clarifies the impact of the molecular weight and structure–activity relationships of antioxidants on their activity. Secondly, this article summarizes and analyzes the extraction of antioxidant peptides from various fish and fish by-products. Through analysis and comparison, it is concluded that fish viscera is more suitable for extracting bioactive peptides. Compared with previous articles that focused on the discussion of the antioxidant properties of fish meat or fish skin extracts, or only paid attention to the antioxidant properties of a single fish by-product, there was no systematic comparison of the antioxidant capacity of extracts from different parts of the fish. Only by judging the activity of antioxidant peptides and the extraction rate can we determine whether a fish by-product is suitable for extracting antioxidant peptides, without comprehensively analyzing the actual processing technology and utilization. This article contributes to a deeper understanding of the structure of antioxidant peptides and the extraction of antioxidant peptides from fish processing by-products, assisting researchers in better developing bioactive peptide segments beneficial to the body from fish processing by-products. This not only expands the avenues for humans to access high-quality and functional proteins but also enhances the utilization of fish resources and mitigates environmental pollution caused by the discarding of by-products.

There are several challenges that must be addressed. Firstly, fish by-product protein hydrolysates often exhibit a fishy odor, which can be undesirable in many applications. Currently, research on effective methods for removing this odor is limited, necessitating further investigation to identify suitable techniques. Secondly, studies have demonstrated that the activity of antioxidant peptides is closely related to their unique amino acid composition. The environmental conditions and biological enzymes encountered during digestion may alter the sequence of these peptides, potentially compromising their functionality. Therefore, conducting animal experiments to specifically target the biological functions of antioxidant peptides is crucial to clarify their in vivo efficacy and mechanism of action. Finally, research has shown that antioxidant peptides can be extracted from fish viscera. However, compared to fish meat and other fish by-products, the structure and composition of fish viscera are more complex. The safety assessment of antioxidant peptides extracted from fish viscera is not yet sufficient, and more toxicological and safety experimental data are needed for support.

## Figures and Tables

**Table 1:** The research status of antioxidants derived from fish by-products.

Source	Amino Acid Sequence	Enzymatic Hydrolysis Method	Antioxidant Property (In Vitro)	Characteristics	References
Large yellow croaker (*Pseudosciaena crocea*) scales	QRPPEPR (879.4 Da) QKVWKYCD (1070.4 Da) VGLPGLSGPVG (952.5 Da)	Hydrolysis with 3.0% E/S (enzyme/scale) of alcalase at 54 °C for 2.3 h.	Eliminating DPPH The smaller the molecular weight, the higher the antioxidant activity.	DPPH RSA: 59.1%	[10]
Skipjack tuna (*Katsuwonus pelamis*) scales	DGPKGH (609.61 Da) MLGPFGPS (804.92 Da)	Enzymatic hydrolysis with 2 g/100 g alcalase at pH 8 and temperature 50 °C for 4 h.	Eliminating ·OH, DPPH, O_2_^−^· The fragment TGH-I with the lowest molecular weight (<3 kDa) exhibits the strongest activity. The Gly, Glu, Asp, Lys, and 2Gly in the amino acid sequences of TGP5, TGP7, and TGP9 may play significant roles in their free radical scavenging activity.	DH: 25.35 ± 1.68%; ·OH RSA: 29.46 ± 1.37% 80.51 ± 3.05% (TGP5) 85.66 ± 2.68% (TGP7) 82.41 ± 2.34% (TGP9)	[11]
Tilapia (*Oreochromis niloticus*) skin	EGL (317.33 Da) YGDEY (645.21 Da)	The optimum hydrolysis conditions for properase E and multifect neutral were 4.5 h, pH 9.0, E/S 5%, and 55 °C and pH 8.0, 4.5 h, E/S 5%, and 35 °C, respectively.	Eliminating ·OH The high antioxidant activity of EGL may be attributed to the presence of Leu at the C-terminus. Metal-chelating amino acid residues Glu and Asp were detected in YGDEY, and its high hydroxyl radical activity may be related to the presence of Tyr at the C- and N-terminals.	DH: 18.01% and 12.60% IC50 value: 4.61 and 6.45 μg mL^−1^	[12]
Horse mackerel (*Magalaspis cordyla*) skin	NHRYDR (856 Da) GNRGFACRHA (1101.5 Da)	According to an enzyme-to-substrate ratio of 1/100 (*w*/*w*), first pepsin is used for enzymatic hydrolysis under conditions of pH 2.5 and 37 °C, followed by the addition of trypsin and α-chymotrypsin for continued enzymatic hydrolysis at pH 8 for 2.5 h.	Eliminating ·OH, DPPH, chelating metal ion	DPPH RSA: 56.4 and 36.3%	[13]
Croaker (*Otolithes ruber*) skin				DPPH RSA: 65.3 and 41.2%	
Bigeye tuna (*Thunnus obesus*) muscle	H-LNLPTAVYMVT-OH (1222 Da)	The mixing of 100 g of substrate with 1 g of enzyme in a 10-L reactor, stirred and cultured for 8 h at the optimal temperature for each enzyme. (a-chymotrypsin, Neutrase^®^ [Novo Nordisk Co., Bagsvaerd, Denmark], papain, pepsin, and trypsin)	Eliminating ·OH, DPPH, O_2_^−^·, inhibiting lipid oxidation, and effectively scavenging intracellular free radicals The presence of Pro, Leu, Ala, and Tyr has a strong scavenging effect on free radicals.	DH: 74.4–80.9%; DPPH RSA: 40.1 ± 1.9 μM	[14]
Monkish (*Lophius litulon*) muscle	EDIVCW (763.82 Da) MEPVW (660.75 Da) YWDAW (739.75 Da)	Hydrolysis with pepsin at 37 °C, with a total enzyme dosage of 1%, is carried out for two hours. Subsequently, the pH is adjusted to 7 with a 1M NaOH solution, and then further hydrolysis is performed with trypsin at 37 °C, with a total enzyme dosage of 1%, for an additional 2 h.	Eliminating ·OH, DPPH, O_2_^−^· Enhancing the endogenous antioxidant enzyme defense system and inducing HepG2 cells to escape from oxidative stress.	DH: 27.24 ± 1.57%; DPPH RSA: 44.54 ± 3.12% ·OH RSA: 41.32 ± 2.73%	[15]
Grass carp (*Ctenopharyngodon idellus*) muscle	VAGW (431.22 Da) APPAMW (671.31 Da) LFGY (498.25 Da) FYYGK (676.32 Da) LLLYK (648.42 Da)	Hydrolysis with protamex (10,000 U/g) at pH 8.0, 50 °C for 3 h, followed by hydrolysis with alcalase (6000 U/g) at pH 9.0, 50 °C for 2 h, and the protein– liquid ratio was 4%.	Eliminating DPPH, ABTS^+^ The VAGW exhibits the highest activity, and the C-terminal amino acid Trp plays a significant role in the synergistic effect.	ABTS RSA: 139.77 μmol GSH/g	[9]
miiuy croaker (*Miichthys* *miiuy*) muscle	YASVV (739.88 Da) NFWWP (569.64 Da) FWKVV (611.66 Da) TWKVV (625.73 Da) FMPLH (1092.23 Da) YFLWP (527.58 Da) VIAPW (578.67 Da) WVWWW (831.98 Da) MWKVW (559.53 Da) IRWWW (574.64 Da)	Hydrolysis by trypsin at pH 8.0, 50 °C, neutrase at pH 7.0, 60 °C, pepsin at pH 2.0, 37 °C, alcalase at pH 8.0, 50 °C, and papain at pH 7.5, 50 °C with total enzyme dose of 1.5% for 5 h.	Scavenging free radicals, reducing oxidative stress, and inhibiting lipid peroxidation Phe, Trp Val, and Lys in the AA sequence of MP3 (FWKVV) and Phe, Met, Pro, Leu, and His in the AA sequence of MP5 (FMPLH) should play a crucial role for their antioxidant capabilities.	DPPH RSA: 52.28 ± 2.80% (papain); 44.15 ± 2.36% (alcalase); 40.86 ± 2.31%(neutrase); 38.75 ± 1.56% (trypsin); 32.76 ± 1.48% (pepsin)	[16]
Skipjack tuna (*Katsuwonus pelamis*) head	WEPPR (683.34 Da) VEE (375.39 Da) WMFDW (783.90 Da) DAGPYGPI (788.90 Da) WMGPY (652.79 Da) ERGPLGPH (861.95 Da) EMGPA (503.58 Da)	First, hydrolyze with pepsin at 37.0 ± 2 °C and pH 1.5, using a protease dosage of 1 g pepsin/100 g defatted powder for 2 h, then adjust the solution pH to 7. Next, hydrolyze with trypsin for 2 h and increase the solution temperature to 95 ± 2 °C for preservation for 10 min.	Scavenging free radicals, reducing power lipid peroxidation inhibition Trp, Met, Phe, Pro, and Ala are important factors that influence the activities of these peptides.	DH: 25.76 ± 1.68%; ·OH RSA: 0.30~2.43 mg/mL; DPPH RSA: 0.31~0.93 mg/mL	[17]
Sardinelle (*Sardinella aurita*) head, viscera	LARL (471.3 Da) GGE (263.08 Da) LHY (431.2 Da) GAH (283.1 Da) GAWA (403.1 Da) PHYL (528.2 Da) GALAAH (538.2 Da)	Under the optimal conditions for each enzyme, the substrate proteins were digested with enzymes at a 0.27:1 (U/mg) enzyme/protein ratio for 3 h. (Alcalase 2.4 L serine-protease from Bacillus licheniformis supplied by Novozymes, crude enzyme preparation from Aspergillus clavatus ES1, alkaline proteases from B. licheniformis NH1, and crude enzyme extract from viscera of sardine)	DPPH radical-scavenging activity The highest activity of Leu-His-Tyr may be attributed to the presence of both His and Tyr residues with its structure.	DH: 5~11%; DPPH RSA: 53.76 ± 1.2%	[18]
Horse mackerel (*Magalaspis cordyla*) viscera	ACFL (518.5 Da)	First, pepsin is used for enzymatic hydrolysis under conditions of pH 2.5 and an enzyme-to-substrate ratio of 1/100 (*w*/*w*) at 37 °C for 2 h, followed by the addition of trypsin and a-chymotrypsin at the same ratio for an additional 2.5 h of enzymatic hydrolysis.	Eliminating ·OH, DPPH The antioxidant activity is higher than that of tocopherol, and the presence of hydrophobic amino acid residue Leu has a significant positive inhibitory effect on lipid peroxidation.	DPPH RSA: 89.2%; ·OH RSA: 59.1%	[19]
miiuy croaker (*Miichthys miiuy*) viscera	FYKWP (739.88 Da) FTGMD (569.64 Da) GFEPY (611.66 Da) YLPYA (625.73 Da) FPPYERRQ (1092.23 Da) GFYAA (527.58 Da) FSGLR (578.67 Da) FPYLRH (831.98Da) VPDDD (559.53 Da) GIEWA (574.64 Da)	Hydrolysis with alcalase at time 3.5 h, temperature 55 °C, pH 9.5, solid–liquid ratio 1:5, and enzyme dose 2.5%.	Eliminating ·OH, DPPH, O_2_^−^·, inhibiting lipid peroxidation GIEWAH (574.64 Da) exhibits higher activity than FPYLRH (831.98 Da). The hydrophobic amino acid residues and aromatic amino acid residues in FPYLRH have a positive impact on its antioxidant activity, while those in GIEWAH contribute to scavenging free radicals and inhibiting lipid peroxidation.	·OH RSA: EC_50_ 0.68 mg/mL and 0.71 mg/ mL for FPYLRH and GIEWA, respectively; DPPH RSA: EC_50_ 0.51 mg/mL and 0.78 mg/mL for FPYLRH and GIEWA, respectively; O_2_^−^ RSA: EC_50_ 0.34 mg/mL and 0.30 mg/mL for FPYLRH and GIEWA, respectively	[20]

## Data Availability

The original contributions presented in the study are included in the article, further inquiries can be directed to the corresponding author/s.
